# “The problem is not detection, it is treatment”: Exploring Why Women Needing Pre-Cervical Cancer Care are Lost to Follow-Up at the Hospital in Iquitos, Peru

**DOI:** 10.21203/rs.3.rs-5059371/v1

**Published:** 2024-12-13

**Authors:** Alex C. Blum, Rachael Tessema, Lauren Nussbaum, Cristina Hidalgo, E. Jennifer Ríos López, Graciela Meza-Sánchez, Rachel Morse, Joanna Brown, Reyles Rios Reategui, Lucía Wong, Luis Díaz Córdova, Karina Gonzales Diaz, Renso López Liñán, Javier Vásquez Vásquez, J. Kathleen Tracy, Valerie A. Paz-Soldan

**Affiliations:** Tulane School of Medicine; Tulane University School of Public Health and Tropical Medicine; Tulane University School of Public Health and Tropical Medicine; Asociación Benéfica PRISMA; Asociación Benéfica PRISMA; Universidad Nacional de la Amazonía Peruana; University of Barcelona; Asociación Benéfica PRISMA; Universidad Nacional de la Amazonía Peruana; Hospital Iquitos; Hospital Regional de Loreto; Gerencia Regional de Salud de Loreto; Universidad Nacional de la Amazonía Peruana; Universidad Nacional de la Amazonía Peruana; University of Vermont College of Medicine; Tulane University School of Public Health and Tropical Medicine

**Keywords:** Loss to follow-up, HPV, cervical cancer, hospital-level care

## Abstract

**Background:**

The objective of this study was to investigate the barriers to follow-up of women with cervical lesions suspicious of cancer who were ineligible for primary-level treatment and needed, but did not receive, hospital-level care in Loreto, Peru.

**Methods:**

In-depth, semi-structured interviews were conducted with 18 HPV-positive women requiring hospital-level follow-up care for cervical lesions suspicious of cancer but for whom there was no documentation of completion of treatment. After thematically analyzing these patient interviews, interview findings were presented to seven doctors and five nurse-midwives at both the hospital and the primary levels for comments and suggestions regarding barriers to treatment. Finally, all findings were presented at a group model building workshop with 19 health authorities and professionals, where action items were discussed to elicit suggestions for systems-level changes.

**Results:**

Interviewed patients and healthcare professionals expressed the need to improve communication between levels of care, as well as the need to implement a patient navigation program. Interviewees also discussed the barriers patients encountered in obtaining hospital-level follow-up that ultimately affected their continuum of care. Patient-identified barriers to hospital care were grouped into three main themes: (1) limited patient understanding of treatment steps, (2) the need for multiple trips to the hospital to obtain care, and (3) lack of provider follow-up, leading to their suggestion for (4) the need for patient navigators. The healthcare professionals concurred with the barriers identified by the patients, but further elucidated suggestions for change such as (1) prioritizing patients with a high risk of cancer when referring to the hospital, and (2) increasing communication between the different levels of care. The group model building workshop served as a space to discuss findings and action items that could potentially make these changes possible.

**Conclusions:**

Despite an overall increase in follow-up for HPV-positive women since the implementation of the new HPV-based screen-and-treat program, women at high risk of developing cervical cancer are still being lost to follow-up after being referred to the hospital for care. The challenges faced by both providers and patients are complex and require systems-level improvements.

## Background

Despite cervical cancer being the fourth most common cancer among women worldwide, prevention and control of this disease is feasible. Many high-income countries have substantially reduced cervical cancer mortality rates, attributing their success to human papillomavirus (HPV) vaccinations [1, 2, 3], high HPV screening rates [4], and timely treatment for those who need it [5]. However, levels of success vary, with many low- and middle-income countries (LMICs) facing challenges when trying to attain these same results [6, 7, 8].

In Peru, despite implementation of policies prioritizing cervical cancer prevention since 1998, cervical cancer remains the second leading cause of cancer-related deaths among women, with an estimated mortality rate of 13.8 per 100,000 [9, 10]. In Loreto, Peru’s largest state that is located within the Peruvian Amazon, the cervical cancer mortality rate is the highest in the country and is 2.6 times the global average [11].

In 2013, WHO published guidelines to support a new “screen-and-treat” approach for the early detection of cervical cancer [12] to support addressing system level challenges preventing women who had received a positive HPV result from obtaining follow-up care. The premise was to provide early treatment or intervention to those at risk (i.e., HPV positive), rather than monitoring for resolution of dysplasia or HPV positivity, which risks losing contact with them. Screen-and-treat models have been shown to be effective in reducing the incidence and/or mortality of cervical cancer [13]. This “screen-and-treat” approach was successfully implemented in 2019 in primary health facilities that make up the Micro Red Iquitos Sur (MRIS) health network in Loreto, Peru [14]. Although the documented loss to follow-up rate improved from 69.8–30% between 2019–2020 after the implementation of the “screen-and-treat” model, women are still being lost in the system – and mostly, they are being lost when trying to access hospital-level treatment [15].

Previous studies conducted by the Proyecto Precancer research team have documented that women are being referred for hospital-level care but are not reaching or completing that care [14]. The current study focuses on identifying factors associated with loss to follow-up among high-risk women at the hospital level: women who were HPV positive and who were ineligible for primary-level treatment with thermal ablation (e.g., suspicious lesions, inability to visualize transformation zone). The specific objectives of this study were to (1) evaluate barriers to hospital-level care among HPV-positive women who did not meet criteria for ablative therapy at the primary level, and (2) explore healthcare professionals’ recommended solutions to reduce hospital-level loss to follow-up.

## Methods

### Setting:

This study was carried out in Iquitos, the capital of Loreto, the largest Peruvian state. Approximately 67% of Loreto’s population is covered by the *Seguro Integral de Salud*, Peru’s largest public health insurance program, which covers those living in poverty or extreme poverty. While most of the state is served by public health facilities, Loreto has only three public hospitals, with two located in Iquitos and one in the smaller city of Yurimaguas, to the South.

Iquitos, with a population of around 400,000, is the world’s largest city accessible solely by air or water transport. The MRIS, the primary health network in Iquitos, comprises 17 health facilities and serves roughly 127,000 people. As reported in previous papers (e.g., [14, 15]), Proyecto Precáncer and local and national stakeholders launched a new HPV-based screen-and-treat cervical cancer program in 2017 within the MRIS. Approximately 20,000 women aged 30–49 within this network were eligible for this program. As a result of this new screen-and-treat approach, there was a 3-fold increase in the number of women screened at the primary level in addition to a 37.2% increase in the number of HPV-positive women who received ablative therapy for precancerous lesions (or who were referred to colposcopy if ineligible) [14].

Women who tested positive for HPV but were ineligible for ablative therapy due to acetowhite lesions on over 75% of their transformation zone, having malignancy suspicious lesions, or having a transformation zone that was not visible were referred to one of the two regional hospitals for specialist follow-up care.

#### Study Design:

**Figure 1:**
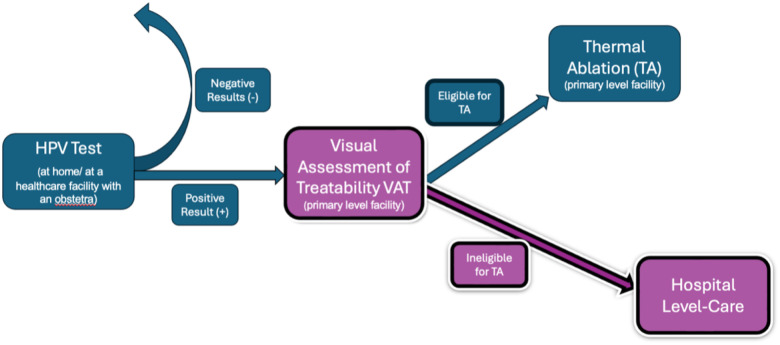
Cervical cancer screen-and-treat model in Loreto.

This study was conducted in three phases. First, semi-structured interviews were conducted with 18 women who were referred to, but did not complete, hospital-level care to explore the reasons for their loss to follow-up. Second, we created a visual depiction of the system and its barriers (Fig. 1) that was used as a discussion point during semi-structured interviews with 12 health professionals working at different system levels to elicit suggestions for change. Finally, all data was presented in a group model building workshop with key government and hospital authorities and health professionals from different levels of care (Table 1).

### Patient Interviews:

#### Sampling and Recruitment:

Women eligible to participate in interviews had received a positive HPV molecular test, were deemed ineligible for ablative therapy at the primary-care facility, were referred to the hospital level for follow-up and had no record of having completed care within three months of their referral date. These women had no record of one or more of the following: (1) attending a hospital level appointment, (2) completing treatment after attending a hospital-level appointment, (3) receiving test results from a hospital-level confirmatory screening (e.g., biopsy). Women were ineligible if they were referred to the hospital for a follow-up and were recorded as having received treatment or a negative confirmatory screening test.

Recruitment began in August of 2022, and continued until September of 2022. Using a generated list of women who had not received hospital level follow up from our registry, we first verified completion information with the nurse-midwives at the primary health facilities, and then by checking for care completion with nurse-midwives at the two hospitals, per the criteria listed above. The research team then called or conducted a house visit to invite eligible women to participate in the study. The goal was to interview 20 women. All approached women agreed to participate in interviews. While 20 women were interviewed, only 18 proved to be eligible, and so the data from the two ineligible women was not included in the overall analysis.

#### Interview Process:

We conducted the interviews in Spanish at the women’s homes or place of work if preferred; two interviews were conducted over the phone because the women were unable to meet in person. The interviews took place in September and October 2022. Consent was obtained prior to the interviews, and the interviews were led by two female Peruvian project collaborators with qualitative experience. The interviews were semi-structured, focusing on issues related to HPV and cervical cancer screening and treatment. Process maps depicting the cervical cancer screening system previously created through interviews and workshops with stakeholders [14] were used to guide the interviews, focusing on the hospital-level of care, including receipt of test results, patient education, the referral process, perceptions about the system – its facilitators and barriers, and general recommendations. In the cases where it was necessary, at the end of the interview, the interviewers provided counseling on HPV and cervical cancer and helped coordinate the woman’s follow-up appointment. All interviews were recorded and transcribed, and field notes were taken to improve understanding but not used for analysis.

### Stakeholder Interviews:

#### Sampling and Recruitment:

Healthcare professionals were eligible for interviews if they were doctors (gynecologists, oncologists, and primary care physicians) or nurse-midwives working in cervical cancer prevention at one of the two hospitals or at the two primary level facilities in Iquitos where ablative therapy occurs. The research team compiled a list of healthcare professionals seeking to include unique roles at different stages of the screen-and-treat program. We recruited 12 collaborators (see Table 1) and all agreed to be interviewed. Recruitment stopped once repetitive themes were identified from stakeholders (“saturation”) at multiple different levels regarding the care trajectory for cervical cancer prevention.

#### Interview Process:

Interviews took place at the participants’ workplaces. All interviews were conducted in Spanish and consent was obtained prior to the interviews. Interviews took place in February 2023, were led by C.H., and were structured around a visual depiction of the system utilizing findings from patient interviews, with six major themes relating to barriers to care (Fig. 1). The interviewer showed Fig. 1 to the participants and proceeded to ask about each process and each barrier, highlighting the specific barriers that were relevant to their own roles and places of work. The interviews also included the healthcare providers’ suggestions for addressing barriers and potential system-level changes. All interviews were recorded and transcribed. Field notes were reviewed but did not inform data analysis.

#### Coding and Analysis:

Both women’s and healthcare professionals’ interviews were coded using mixed inductive and deductive methods. The women’s interviews were coded after completion of all 18 interviews and prior to the initiation of stakeholder interviews. Stakeholder interviews were coded after completion of all 12 collaborator interviews. For the women’s interviews, we used codes that described knowledge of, sentiments toward, and major obstacles at each level of care. These codes were analyzed to understand barriers to care, but also included suggestions and recommendations for change made by the women. For the stakeholder interviews, we created a codebook which grouped stakeholders’ suggested changes into individual-level and systems-level, further subdivided by the specific barrier the change was aimed to address. Stakeholder interviews were analyzed to identify suggestions for change, but barriers were also identified and coded. Two researchers with coding experience read through interviews coding deductively and creating new codes as needed using Dedoose software. The coders met frequently to discuss the codes to ensure consistency; for both the women and collaborator interviews, five interviews were read through and discussed prior to completion of the final codebook. From thereon, one researcher coded the remainder of the interviews.

## Group Model Building Workshop

### Recruitment:

Key stakeholders in the cervical cancer health system within the MRIS were identified by members of the Proyecto Precancer team, along with the cancer coordinator of the Ministry of Health. The 19 invited parties included: doctors and nurse-midwives working in the gynecology and oncology departments at the two hospitals, referring primary-care facilities, and various rural primary-care facilities; hospital administrators and regional health authorities.

### Workshop Process:

Data from the women and collaborator interviews was organized and visually represented in graphs and charts. This was used to generate discussion during the workshop about the findings and the suggestions that emerged from the interviews.

Following the presentation, stakeholders were divided into two groups based on where they worked: primary or tertiary level. Each of the groups was facilitated by two Proyecto Precancer team members to reach consensus on what could be done to improve the system, by whom, and how. Participants created diagrams which were then documented in photos, action items were determined, and a commitment from those present to make the changes was agreed on.

## Results

Four major themes emerged from the interviews with patients and health care professionals, which were then discussed in the workshop: a limited understanding about HPV and the next steps of care among women, having to make multiple trips to the hospital for follow-up (including difficulty making appointments and receiving care), the lack of follow-up from health care professionals (including difficulty obtaining results), and the need for a patient navigator.

### 1. Limited Understanding of their Disease and the Next Steps of Care:

A major and repeated theme was that women had a limited understanding of their HPV diagnoses and the next steps of care needed when referred to the hospital-level for treatment. They expressed uncertainty regarding what exactly it meant to be HPV positive and/or ineligible to receive care at the primary level. The confusion and frustration women described also led to fear and distress: *“I thought I was going to die; I spent some days thinking I was going to die.”*

**Figure 2:**
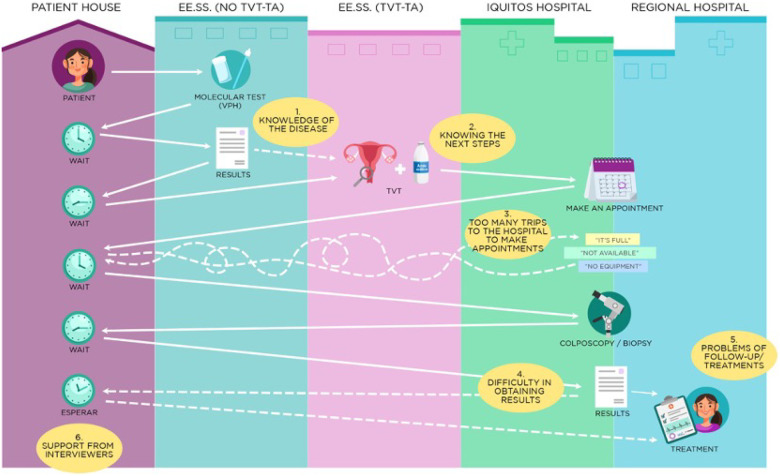
Visual Depiction of System Barriers from the Interviewed Women’s Perspective

The non-standardized trajectory of referrals also led to confusion, which perpetuated distress (see Fig. 2). Women who are HPV positive and require hospital level follow up are referred to either the gynecology unit at one of the hospitals or the oncology unit of the other; this can lead to confusion about the possibility they had cancer, or as one woman referred to oncology described:

In oncology, I always knew that’s where people go with cancer, and I felt that I was sick, and I felt nervous and scared because one time I went there with a friend to get her mother’s results who had cancer, and she told me that there [the oncology unit] is where people go with advanced cancer

This lack of knowledge persisted upon physically reaching the hospital, as many women described confusion regarding which department to visit and what to expect when finally seeing a provider. This confusion seemed to stem from a disconnect within the hospital system and the lack of clear policies at admissions about where to make the appointments: *“They said to me [at gynecology], ‘How are they going to refer you to oncology if you don’t have cancer? You should have come to us.”*

The communication challenges within and between the primary and tertiary levels of care was discussed as part of each healthcare professional interview as well. In addition to communication challenges, healthcare professionals also identified high turnover rates of medical personnel and staff at all levels of the healthcare system as a barrier to successful engagement of women for treatment follow-up. Further, it was suggested that medical personnel at times do not have a holistic or current understanding of the new screen-to-treat program, and some are not aware that the current best clinical practices have shifted from Pap smears to molecular HPV testing that is either self-administered or performed by a healthcare professional:

In Loreto, doctors of any specialty that is not cancer do not know anything about cancer. They only know what cancer is, nothing more. Given that, if doctors that do not have anything to do with oncology don’t know anything about cancer, imagine the knowledge level for the general population. So we have a lot of work to do in terms of training, education, re-structuring the levels of care to be able to grow and better manage this pathology.

### 2. Multiple Trips to the Hospital for Follow-Up (Difficulty Making Appointments and Receiving Care)

Another dominant theme was that while most women were eventually able to figure out how to make an appointment at the hospital through the guidance of nurse-midwives at the primary level, this process proved to be a challenge. They attributed this to a lack of familiarity with the appointment-making process (which has to be in person), miscommunication from providers about where to go or who to ask for, and the lack of an organized system in place to support them in making these appointments (i.e., in one hospital, they get seen in gynecology, in the other at oncology). Not a single participant interviewed was able to receive care upon arrival at the hospital on her first visit, and over half had to make at least three trips to the hospital before receiving care:

The fourth time I came back, I got the appointment. [The first time] they told me that even though the doctor was there, he had to operate [and was unavailable]. They told me to come the next day. Then the next day, [I went but] the doctor said he wasn’t coming, he’s coming tomorrow. And the next day, I went and they were going to see me, but the appointment that I had supposedly made was not made, so he wasn’t there. The doctor was on vacation.

The women stated that the main reasons they had to make multiple trips to the hospital was because their doctors were not available on scheduled days due to the doctor being away (e.g., last minute training or event), their schedule being overbooked, or the doctor or other healthcare professionals having been on strike.

Women expressed that each additional trip to the hospital led to a loss of time and money that they did not have, and that they needed to prioritize caring for their families. Some women felt angry with the system for functioning in an inefficient manner that forced them to wait for care while their disease continued to advance.

During the interviews with the health professionals, it became evident that many sympathized with the difficulty and time-consuming nature of making appointments, receiving care, and navigating the healthcare system:

Unfortunately, I think that we have a failed system, an inefficient system that does not work, that is not responding to demand, and unfortunately this is not just at the hospital level, but starting at the primary level. Before, you could go to a health post or a health center, get in line, and you would be seen that same day. Now, you have to go to a health center as if it were a hospital and they tell you “we have appointments in two weeks,” so the supply is very little. But the issue is not just supply, but also the health centers’ logistical capacity is very poor. So, if we do not improve that capacity, if we do not expand supply, the system will continue to collapse.

### 3. The Lack of Follow-Up from Health Care Professionals (Including Difficulty Obtaining Results)

The third common theme was that after the women had been seen by a provider at the hospital and had undergone diagnostic tests, such as a colposcopy or biopsy, many of the women then described encountering difficulties with getting their results or not being contacted for follow-up. The women stated that they had expected to receive a call about their results and never did. Some described a general fear about not knowing their results, leading to fearful thoughts about what was going to happen to them and their families.

I felt bad because they told me I have cancer, that I will die early… I was so upset that I stopped going to the health post, what can I do, if so much time has passed and they didn’t call me [for follow-up], they wasted the whole month, they treat me like an animal, it’s not right, but I got my treatment, I went to the regional hospital.

Many of the women stated that with time, they developed feelings of indignation and hopelessness. When one of the participants was asked about her feelings during the interview, she said that she was surprised that anyone had contacted her at all because the interviewer was the first person she had heard from since going to the hospital to receive additional testing after receiving her initial positive HPV-molecular test at a health center,

Another common concern that arose was that of dual practice among the nurse-midwives and doctors; several women mentioned being referred by a nurse-midwife to her own private practice to receive treatment. They discussed that a nurse-midwife would require such visits before she would schedule an appointment with the hospital doctor, and as a result, these women were not receiving their follow-up care. In a few instances, women resorted to natural medicines or other medications that the nurse-midwives were promoting as helpful for their condition.

Overall, women expressed that they felt that the formal health system had abandoned them.

I went to the health post because I had to go there to make my appointment [for treatment] for the end of July, but when I arrived, the midwife told me that they couldn’t do it because the machine was broken, but that they would let me know. So I told her, “I will come back at the end of September.” She told me “Okay,” but they still haven’t called me, they haven’t even given me my results, because the midwife told me they were going to bring some new equipment, I don’t know what.

All 12 healthcare professionals acknowledged the patients’ difficulties in coordinating their appointments and care on their own. Health professionals confirmed that this is in part due to insufficient coordination between hospital units, as well as a non-existent patient registry system. The idea of maintaining a patient registry that can be shared between the primary and tertiary levels of care was discussed as a potential solution to the communication challenges between healthcare sites and levels. The health professional interviewees suggested that the registry should include information such as the types of tests performed, the results, follow-up appointments attended, treatment recommended and received, etc. This would be useful to all providers in managing a patient’s care, and if a patient navigation program was implemented, could be used by this individual as well.

### 4. The Need for a Patient Navigator

Toward the end of all 18 patient interviews, the interviewers provided education and counseling on HPV, cervical cancer, and the continuum of care in Iquitos. If any of the women needed a follow-up appointment, the interviewers coordinated it and gave them instructions on where to go, how to get there, and when to do so. All women interviewed expressed that assistance like this throughout their care trajectory would be very useful. One participant stated:

It would be easier to have a designated member of the health facility, and someone there that would know if and when the patient had to come to each facility. It would also be helpful if there was someone, maybe a health professional, who we could call and follow up with. This would be helpful because then the patient would not have to do all that.

At the end of the interviews with health professionals, they were asked what advice they would give to relatives or friends navigating this system, and they suggested the potential benefits of having someone in the healthcare system whose role it is to help patients communicate with providers and navigate each step of the system. Alternatively, most felt it would be beneficial for patients to have a friend or relative with them during the counseling process, which included discussing results and the next steps of care – with the idea that an extra set of ears would help.

It would help if the health center had a phone number that patients could call to know if they have to go back to the heath center, or if there was a health professional that they could call for follow-up. That way the patient would not have to do all of this, to travel to the hospital, wait up to six months and still not have their test result. Because what are you supposed to say to the patient? Once you get your biopsy, come back in six months? I can’t say that. That’s not a viable option. There should be a better solution. There should be a phone number to call, that’s my recommendation, so that after the doctor sees you, if there is follow-up pending or you have to make another appointment, you call that number and they tell you if they will be able to see you.

## Suggestions for Change

The four identified themes discussed above were presented at the group model building workshop with health professionals, where professionals were divided into two groups to define ways to address some of the identified challenges and bottlenecks. Suggestions were discussed thoroughly, with key action steps defined before the professionals left.

### 1. Prioritization of Patients with a High Risk of Cancer

The health professionals all agreed that HPV-positive women who are ineligible for primary-level treatment due to suspicion of cancer need to be prioritized when referred to the hospital. Suggestions included updating the referral form for these patients, to include an “urgent box” that can be checked, or asking the provider making the referral to call one nurse-midwife directly to ensure that patient was prioritized at the time of making an appointment. This latter suggestion was shared among all present to ensure a do-able action that could be carried out immediately since all key stakeholders were at the workshop. An additional suggestion was to increase education among all hospital workers about the “screen-and-treat” model to ensure they understood that women being referred had been seen by a primary level doctor due to the positive HPV test and required priority appointments.

As this topic was explored, hospital professionals discussed the speed at which they could intervene if needed. Initially, the oncologist mentioned being able to operate on urgent cases within days. Further clarification led to the reflection that, prior to any surgical intervention, the patient needed to be cleared by cardiology and obtain some blood tests, and that sometimes there are delays involved with these additional steps. An action step decided on was that the head of oncology would speak to the head of cardiology to find ways to speed up pre-surgical appointments.

### 2. The Utility of Patient Navigation

Multiple models for patient navigation were discussed at the workshop based on systems that the healthcare professionals had seen in other departments. The models included using volunteers and healthcare workers as navigators who could provide education, advice, and logistical plans for patients in terms of next steps of care, and the possibility of having a health professional running a telephone line to track and follow up with patients.

The role of a patient navigator was further compared to the role that the Proyecto Precancer interviewer had played during the interviews with the women where, in all cases, they provided information on HPV and cervical cancer and helped coordinate the women’s next follow-up appointments. This led to a discussion about a patient navigator that worked helping indigenous people from remote areas of the rainforest navigate the hospital system when needed. This patient navigator would become involved when there was a referral from an indigenous community where individuals are less connected to the health system and may not speak Spanish. Discussion of this topic within the group model building workshop led both healthcare professionals and hospital administrators to set an action item to speak with their colleagues and understand who paid for the patient navigator at the moment, and to explore the feasibility of onboarding patient navigators.

### 3. Increasing Communication between Levels of Care

Many collaborators expressed the importance of the referral process being directly from one healthcare professional to another to ensure that each of the levels of care has some awareness of the referral. They described how this would be more effective than sending the patient with a slip of paper without notifying the practitioner that they were sending them to. WhatsApp groups were also highlighted as an effective way for nurse-midwives to communicate between the different levels of care.

In the group model building workshop, stakeholders from all levels of care discussed the issue of a lacking patient registry, making patient management impossible. One of the health authorities present who was familiar with the REF-CON (*referencia y contrareferencia*) system described that it was designed to assist providers in patient follow up. The REF-CON system is meant to be used by primary level providers to send patients to tertiary level care (“REF”), and then by tertiary level providers to inform back to the primary level what was done and the needed follow up (“CON”). This sub-group decided it would be more efficient to pilot this system to evaluate its effectiveness for the cervical cancer prevention and control system, than to try to create a new system. One doctor recommended adding a feature that would create an alert or notification for the midwife when a patient did not start or continue treatment, so that the midwife could contact the patient to reschedule her appointment.

## Discussion

As previously noted, four main themes emerged as the major barriers that women experienced first-hand to accessing care at the tertiary level: (1) limited understanding of their disease, (2) difficulty making appointments and getting care, (3) a lack of follow-up from healthcare professionals, and (4) the lack of a patient navigation system. Based on these identified barriers, collaborator interviews with 12 healthcare professionals were used to elicit suggestions for change. These professionals suggested prioritizing patients with a high risk of cancer, using a patient navigation system, and improving communication between levels of care.

Many of the barriers identified have also been described in other studies. LMICs like Rwanda, Kenya, and Ethiopia all struggle with high loss to follow-up for women with HPV or cervical cancer due to factors like long travel distances, lack of patient tracking systems, inadequate patient navigation, poor understanding of HPV, transportation costs, household obligations, fear of treatment, and delays in medical services. Each country has specific barriers, including decentralized healthcare in Rwanda, cultural and logistical challenges in Kenya, and delays and costs in Ethiopia [16, 17, 18]. Missing from the literature, however, is any investigation into the challenges and factors that are contributing to the loss to follow-up at the hospital-level among HPV-positive women in Latin America. This study aims to fill this gap in the literature by providing insights in the context of Loreto, Peru.

Based on the findings discussed above, we have identified four major implications at the program, policy, and research levels. First, implementing health literacy policies and training programs for providers would address the women’s lack of a clear understanding of their disease and the healthcare system. These trainings would teach providers how to convey information in a way that matches their patient’s health literacy level. This could include visual tools, such as a flowchart with instructions of the patient’s next stage of care. Health literacy trainings for physicians has improved health outcomes for patients in several different countries, from the United States [19] to Iran [20]. Health literacy training programs that are tailored to the health care system at hand and that keep in mind the socioeconomic and cultural contexts of the patients are also recommended by the CDC to improve health outcomes [21].

Second, a telephone-based patient navigation system could support women in scheduling appointments, accessing their results, and navigating the healthcare system as a whole. The current system requires that women take the primary level referral to the hospital in person to make an appointment, then go to an appointment, then return to the hospital to pick up a paper copy of their results to make a follow up appointment, and so on. They require this because healthcare providers get compensated from the state using a specific code for “giving out results.” They are not supposed to give out results by phone or text, but this requires multiple visits from patients to handle logistics in person – a task not feasible for individuals who might live hours away from the hospital and do not have the time or money for multiple trips (including the trips where doctors are not available). A new remote health service provided via computers, phones, and other digitized systems, called Telesalud, has been gaining momentum in some of the larger health centers [22]. A potential implication is to create a new code within this system that allows patients to receive their results over the phone while still allowing the healthcare provider or individual providing the service to be compensated for doing so.

Third, in Iquitos, new technology has rapidly integrated into people’s daily lives, where many have access to both phones and the internet. People commonly use platforms like Facebook, Instagram, or WhatsApp to communicate and get their information. These platforms could be an invaluable tool to support HPV-positive patients in navigating the health system in Peru by providing an accessible way to get information while using the technology they already have at their fingertips [23]. On the other hand, the digital divide could lead to even less support for the most vulnerable who do not have consistent access to phones and/or internet connections, or who are not computer literate. To ensure that further inequities do not arise, these mobile-based solutions should supplement, not replace, the current system that requires patients to pick up their results in person. Further research is needed to explore patient navigation models for this population.

The final implication concerns the high turnover rates of medical personnel and staff not having a holistic understanding of the new screen-and-treat system and updated clinical practices. To ensure medical personnel have current information on HPV and its treatment, medical schools must teach the most current information about HPV and its treatment to their students. Furthermore, having virtual onboarding and training systems for new health staff (which are supposed to exist but are not actually put into practice) could be beneficial for efficiently dealing with the high turnover rates. This would also ensure that healthcare professionals have access to updated information, as currently most providers travel to Lima for training purposes.

### Limitations:

This study was conducted in the MRIS, the largest health network in Iquitos, and selected due to its centralized location and its comparatively efficient documentation system. The suggestions for change may not be applicable to other regions of Peru or even Loreto due to differing structural and geographic barriers. However, the findings from this study reveal the need for further research in the region to identify barriers causing loss to follow up in other similar regions, and the need to address these. Furthermore, many of the women interviewed discussed the barriers they faced when getting care at the hospital in the context of COVID-19. While COVID-19 was unprecedented and may have caused further fragmentation of care, we were unable to determine which, if any, barriers were heightened by COVID-19. Despite these limitations, major patterns emerged in our interviews and workshop regarding barriers to accessing tertiary level care and future suggestions for change.

## Conclusion

Despite an increase in follow-up for HPV-positive women overall since implementation of the new HPV-based screen-and-treat approach in Loreto, women who are at a higher risk of developing cervical cancer are still being lost after their referral to the hospital level for care. Factors associated with the loss to follow-up included the lack of understanding of next steps of care and their disease, the need for multiple patient trips to the hospital for appointments and care, the lack of standard follow-up processes from providers, and issues with receiving results (for both patients and providers). System improvements should focus on developing an integrated registry that can be accessed by primary and tertiary level providers and testing and implementing a patient navigation system for women.

## Figures and Tables

**Table 1:** Participant Demographics

Role	Number of Individuals
Phase 1: Patient Interviews	
HPV-Positive Women Lost to Follow-Up	18
Phase 2: Collaborator Interviews	
Primary-Level Physician	1
Primary-Level Midwife	2
Hospital-Level Physician	6
Hospital-Level Midwife	3
Phase 3: Group Model Building Workshop	
Primary- Level Midwife	4
Primary- Level Physician:	5
Hospital-Level Midwife	1
Hospital-Level Physician	4
Administrative Assistant	1
Nurse	2
Regional Health Authority	2

## Data Availability

The dataset supporting the conclusions of this article is available upon reasonable request and with the participants’ permission, pursuant to the terms of the IRB approval.

## Abbreviations

LMICs: low- and middle-income countries
HPV: human papillomavirus
MRIS: Micro Red Iquitos Sur
REF-CON: *referencia y contrareferencia*
